# A Crisis-Responsive Framework for Medical Device Development Applied to the COVID-19 Pandemic

**DOI:** 10.3389/fdgth.2021.617106

**Published:** 2021-03-22

**Authors:** Marc-Joseph Antonini, Deborah Plana, Shriya Srinivasan, Lyla Atta, Aditya Achanta, Helen Yang, Avilash K. Cramer, Jacob Freake, Michael S. Sinha, Sherry H. Yu, Nicole R. LeBoeuf, Ben Linville-Engler, Peter K. Sorger

**Affiliations:** 1 Greater Boston Pandemic Fabrication Team (PanFab) c/o Harvard-MIT Center for Regulatory Science, Harvard Medical School, Boston, MA, United States,; 2 Research Laboratory of Electronics, Massachusetts Institute of Technology, Cambridge, MA, United States,; 3 McGovern Institute for Brain Research, Massachusetts Institute of Technology, Cambridge, MA, United States,; 4 Harvard-MIT Division of Health Sciences and Technology Program, Cambridge, MA, United States,; 5 Department of Systems Biology, Harvard Ludwig Cancer Research Center and Harvard Medical School, Boston, MA, United States,; 6 Department of Mechanical Engineering, Massachusetts Institute of Technology, Cambridge, MA, United States,; 7 Division of Gastroenterology, Hepatology and Endoscopy, Brigham and Women’s Hospital and Harvard Medical School, Boston, MA, United States,; 8 David H. Koch Institute for Integrative Cancer Research, Massachusetts Institute of Technology, Cambridge, MA, United States,; 9 Department of Biomedical Engineering, Johns Hopkins University School of Medicine, Baltimore, MD, United States,; 10 Harvard Medical School, Boston, MA, United States,; 11 Harvard-MIT Center for Regulatory Science, Harvard Medical School, Boston, MA, United States,; 12 Fikst Product Development, Woburn, MA, United States,; 13 Department of Dermatology, Yale University School of Medicine, New Haven, CT, United States,; 14 Department of Dermatology, Center for Cutaneous Oncology, Brigham and Women’s Hospital, Dana-Farber Cancer Institute, Boston, MA, United States,; 15 System Design and Management, Massachusetts Institute of Technology, Cambridge, MA, United States,; 16 Massachusetts Manufacturing Emergency Response Team (MA M-ERT), Massachusetts Technology Collaborative, Westborough, MA, United States

**Keywords:** personal protective equipment (PPE), COVID-19, manufacturing, prototyping, biocompatibility, 3D printing, regulatory sciences, medical device design

## Abstract

The disruption of conventional manufacturing, supply, and distribution channels during the COVID-19 pandemic caused widespread shortages in personal protective equipment (PPE) and other medical supplies. These shortages catalyzed local efforts to use nontraditional, rapid manufacturing to meet urgent healthcare needs. Here we present a crisis-responsive design framework designed to assist with product development under pandemic conditions. The framework emphasizes stakeholder engagement, comprehensive but efficient needs assessment, rapid manufacturing, and modified product testing to enable accelerated development of healthcare products. We contrast this framework with traditional medical device manufacturing that proceeds at a more deliberate pace, discuss strengths and weakness of pandemic-responsive fabrication, and consider relevant regulatory policies. We highlight the use of the crisis-responsive framework in a case study of face shield design and production for a large US academic hospital. Finally, we make recommendations aimed at improving future resilience to pandemics and healthcare emergencies. These include continued development of open source designs suitable for rapid manufacturing, education of maker communities and hospital administrators about rapidly-manufactured medical devices, and changes in regulatory policy that help strike a balance between quality and innovation.

## INTRODUCTION

### Rapid Product Development to Meet Emergent Shortages of Medical Supplies

In the face of a global COVID-19 pandemic, widespread disruption of international supply chains and local distribution networks has led to severe shortages in personal protective equipment (PPE) and other medical equipment such as ventilators (1). These shortages have spurred numerous local efforts to supply alternative products. Such efforts involve a diverse community of scientists, engineers, physicians, hobbyists (the “maker” community), community-based organizations, and industrial manufacturers not previously involved in supplying healthcare products. Numerous international collaborations have been formed, anchored in open-source designs, rapid dissemination of pre-prints (on medRxiv or bioRxiv) and repositories such as the National Institute of Health’s 3D Print Exchange (2). Such non-traditional fabrication of medical equipment is made feasible by the widespread availability and low cost of manufacturing techniques including 3D printing and laser cutting. These approaches are ideal for low-volume production of face shields, masks, frames for N95 filtering facepiece respirators (“N95 masks”), swabs for diagnostic kits, and potentially more complex medical products such as ventilator parts (3, 4). Many of these devices are safety-critical items designed to control infection risk or sustain life. There is therefore reason for concern about medical products that are manufactured using non-traditional methods and supplied by relatively inexperienced fabricators. We nonetheless propose that the capacity for crisis-responsive local manufacturing be further developed so that it can contribute to resilience to pandemics and healthcare emergencies at local, national, and international levels. By analogy, local repair and rebuilding capacities have long been recognized as critical aspects of resilience to natural disasters (5).

### A Crisis-Responsive Design Framework

One of the greatest challenges facing non-traditional producers of medical equipment is the complex and unfamiliar regulatory landscape in place for safety-critical products. Thus, in an emergency setting, design validation and testing—not initial design and final fabrication—are often the biggest barriers to the introduction of new or alternative products. As a consequence, there have been multiple instances in which maker communities or small manufacturers have created a needed product, only to find it turned away by healthcare providers and hospitals (6). The primary goal of this perspective is to prevent such situations by providing an overview of medical device development to makers, engineers, and manufacturers who are not traditionally involved in the medical industry. We also elaborate on the development of design and regulatory frameworks relevant to future emergencies, with a focus on PPE and similar “low-risk” medical devices. We end with some considerations for regulators that could be applied to future pandemics.

Like the traditional framework for medical device development, the crisis-responsive framework outlined here incorporates systems-level interactions among producers and stakeholders that impact product development, testing, and deployment. In a crisis however, it is necessary to reframe a traditionally deliberate, iterative, and highly controlled process for medical device development into a methodology that can be performed on an accelerated timescale with unfamiliar stakeholders and without compromising product safety. Use of a crisis-responsive framework ensures that hospital incident commands, healthcare leadership, institutional review boards (ethics committees), product designers, and fabricators can work efficiently together in pursuit of enhanced resiliency to medical emergencies.

### Comparing Traditional and Crisis-Responsive Design Frameworks for Medical Device Development

**FIGURE 1 |:**
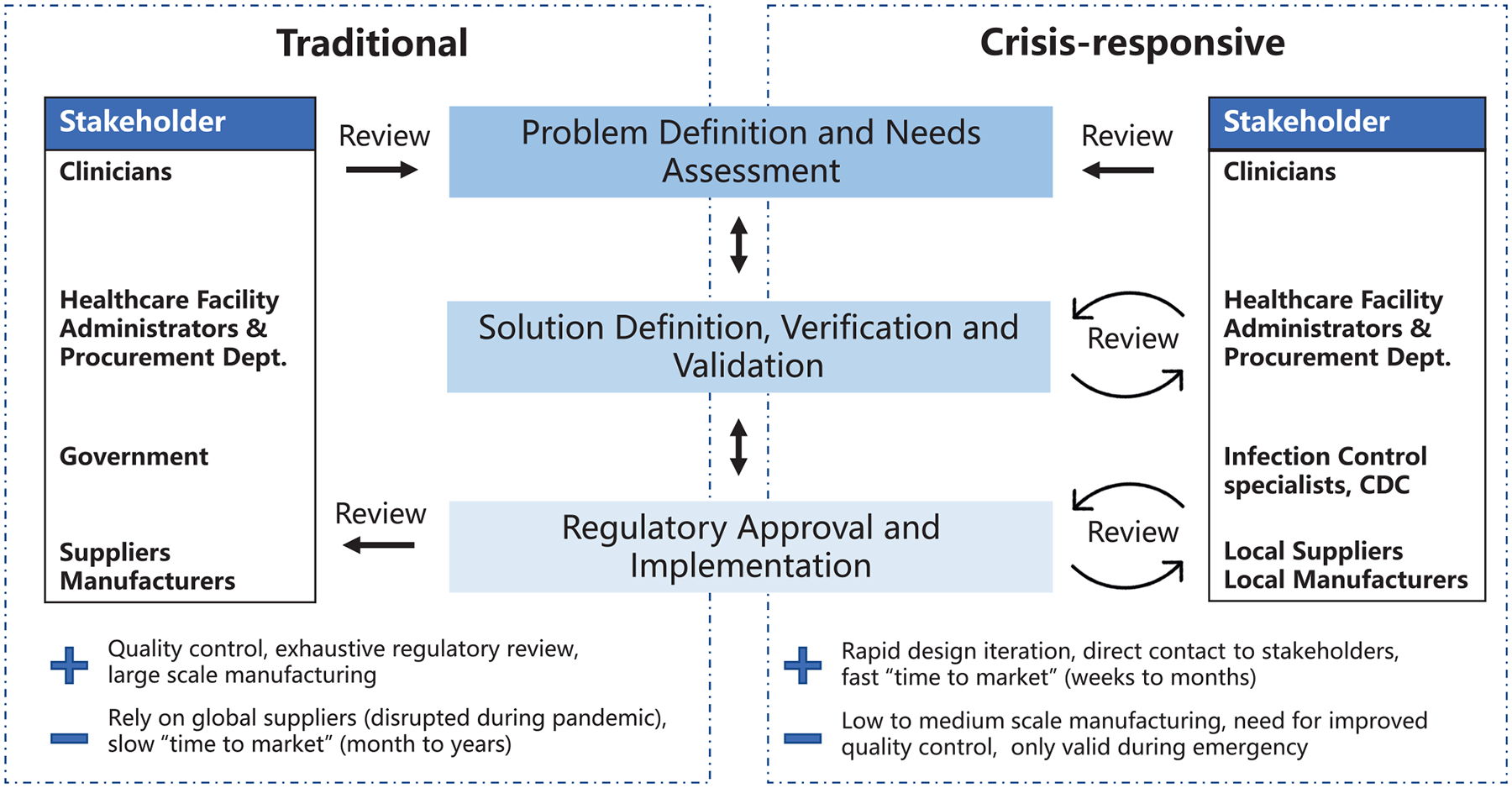
Traditional and crisis-responsive design framework for medical device development. The crisis-responsive framework places emphasis on repeated design review using input from a broad range of stakeholders, all of whom may face unique constraints due to the pandemic.

A variety of models have been developed to describe the different stages of medical device development and their relationships to each other (7–12). Key steps include: (1) problem definition and needs assessment, (2) solution definition, verification, and validation and (3) regulatory approval and implementation (Figure 1). Here we highlight two development models: the traditional waterfall process, first developed in 1970 to describe software development (13) and historically used by most medical device manufacturers, and a crisis-responsive framework, better suited to tackle the rapidly-changing demands of the pandemic. The later framework borrows from “agile product development” (14) and emphasizes flexibility, rapid implementation of new features to respond to changing requirements, and fast delivery of a working product (Figure 1). While the waterfall model emphasizes feedback and iteration primarily at the product validation stages when a design has been fully implemented (15), the crisis-responsive model involves review and iteration at earlier stages in a design; this is essential because it is rarely possible to undertake formal market research or systematic needs assessment under pandemic conditions. From the perspective of time scales, agile development parallelizes steps to the extent possible to eliminate waiting periods. Use of agile product development and rapid manufacturing makes it possible to create finished prototypes on a time scale of days to weeks as opposed to months to years, as in the case of traditional waterfall-type development, facilitating iterative design and testing with end-users. In a healthcare setting this is likely to include senior physicians and hospital leaders who would not normally be involved in PPE selection. Crisis-responsive development relies on the willingness of hospital stakeholders to consider unfamiliar, innovative, and more costly designs based on an assessment of risks posed by the unavailability of traditional products.

In conventional needs assessment, the impact of selling price, access to retail and wholesale channels, and brands are carefully considered; channel access and branding allow commodity products lacking strong intellectual property protection (e.g., face shields, N95 masks, gowns) to sell at a premium price. Nonetheless, the pressure on price is high, and low price margins pose the primary limitation on innovation. In many cases, low margins make domestic production infeasible causing a small number of overseas manufacturers to provide the majority of PPE products. For proprietary products, margins are typically higher and innovation more important, but the willingness of third parties or national healthcare systems to reimburse for a product is a major consideration. Price sensitivity varies among private and public health systems, nursing homes, and independent living facilities, leading to a plethora of functionally similar products distinguished primarily in branding and distribution channels.

In a crisis, however, the prioritization of these concerns is shifted because the goal is typically to produce the best possible product in the shortest amount of time. Given limitations in fabrication facilities, materials, and the skill of the design team, price and branding are deprioritized, particularly for items such as PPE that suddenly become essential and require significant effort for procurement teams to obtain in volume. Similarly, access to retail and wholesale hospital supply channels, which is typically dominated by a small number of large companies, becomes a secondary consideration during a pandemic. This is particularly true because in the COVID-19 pandemic it is precisely the failure of traditional supply chains to meet urgent requirements that has created the need for nontraditional suppliers. These changes fundamentally alter the stakeholder landscape and the design process. Getting products from a fabricator to a customer is still essential, and involves procurement departments, but is made easier when production is local to the customer and the customer is directly engaged in specifying and testing prototypes.

A crisis-responsive framework has many potential limitations in terms of regulatory compliance and sustainability and is not suitable for use under non-emergency conditions; it is intended to provide stopgap solutions to meet immediate needs. Crisis-responsive design typically does not include the documentation needed for regulatory review and often relies on small-scale production. Designs are sensitive to unanticipated substitution of input materials due to supply shortages. Brand identity is rarely considered, and the analysis of intellectual property may be incomplete. Despite these limitations, even in a crisis it is essential that a rational and well-considered process be followed to ensure that products are functional, reliable, and as safe as possible. Only then can manufacturing by local communities help rather than hinder emergency response. Governments also have an important role to play in creating emergency authorizations and temporarily overriding some intellectual property protections.

## STAGE 1: PROBLEM DEFINITION AND NEEDS ASSESSMENT

### The Importance of Stakeholder Input

Traditionally, problem definition involves assessing the needs of end-users or healthcare systems through market research. Alternatively, in a crisis, it is common for designers and fabricators to work directly with end-users, such as healthcare workers, rather than with traditional procurement departments. Health care workers will be most concerned with the usability, reliability, and testing of a product and least concerned with branding and cost. Design, manufacturing, distribution, risk-mitigation, and lifecycle considerations (e.g., sterilization) remain the purview of the design and fabrication teams, but we have found that end-users are often willing to engage in issues of design and fabrication. Notably, direct contact between designers and users provides a rare opportunity for innovation in areas such as PPE, a type of product for which new devices are slow to emerge despite long-known deficiencies in current products.

The process of defining requirements and engaging stakeholders will differ in private and public hospitals, private practices, nursing homes, and independent living facilities but in general, it is end-users who will drive the process. Designers may need to coordinate with individuals empowered by hospital administrators, hospital incident commands (in charge of emergency response) (16), purchasing and procurement departments, and hospital administrators in order to better understand current needs. Local and state government officials can also be a resource for regulatory, purchasing power, and supply chain information and should be consulted if possible. In many cases, non-traditional medical products use components that were manufactured for other purposes (e.g., vacuum cleaner filters for use in PAPRs). Local suppliers and distributors can be an invaluable source of information on the availability of such materials and equipment and their technical performance. We have found that, during the COVID-19 pandemic, many materials suppliers are willing to provide extra help to fabricators who are not part of their traditional customer base. A final and important aspect of needs assessment is soliciting requirements from a diversity of end-users who differ in gender, body size and shape (e.g., differing face dimensions in the context of respirators) and also in clinical roles (e.g., nursing staff, physicians in emergency rooms, outpatient consultants, orderly staff, custodial staff). Non-traditional fabricators must take care to reduce inequities in the workplace and in patient access to health care, not amplify them.

### Coordinating With Multiple Stakeholders

At the outset of the COVID pandemic, many municipalities and even hospitals had their own design and fabrication teams working largely independently of each other, although often using shared designs and methods. Several months into the pandemic, particularly after the first wave of hospitalization passed, local fabrication teams started to work together to improve efficiency and share expertise. State programs such as Massachusetts Manufacturing Emergency Response Team [M-ERT (17, 18)] and national efforts such as America Makes (19) are playing an increasingly important role in matching end-users with suppliers and in providing access to tested designs, materials, and supply chains. Including such groups in the design and fabrication processes can bring substantial benefits in terms of the suitability of the design and feasibility of fabrication.

### Traditional and Nontraditional Supply Procurement

The first stage in meeting urgent supply shortages is to look for alternative medical manufacturers of similar products. When such products are not available, an alternative solution is to find non-medical suppliers of functionally related products and components. For instance, the Greater Boston Pandemic Fabrication Team (PanFab) (20) PAPR design uses commercially available high-efficiency particulate air (HEPA) vacuum filters since supply shortages have made it challenging to procure filters traditionally used in healthcare settings (20). In addition to meeting device shortages, looking “sideways” in the supply chain through the creation of modified products can help meet PPE shortages in novel ways. For instance, many N95 respirators become unusable after several donning and doffing cycles due to poor fit of the nosepiece (the metal tabs often become distorted) and degradation or breakage of the elastic straps that hold masks in place. Because manufacturing N95-type respirators requires highly specialized fabrics and equipment, it is more feasible to fix the problems with existing masks than to make new ones. As a result, multiple groups have developed 3D printed mask frames that can fit over existing N95 masks and take the place of degraded or broken nosepieces and straps (21–27). Like many other innovative products developed to meet emergency needs, mask frames may also have a role in respiratory protection under non-crisis conditions.

### Determining Raw Materials Needs

Needs assessment in a crisis must not only consider the requirements of end-users, but also the capabilities of manufacturers and suppliers. During a pandemic, acquiring raw materials is often challenging, as suppliers may be closed or an entire class of material may be out of stock (e.g., thin BoPET sheets commonly used in face shields). It is therefore important to consider equipment and supply constraints and assess alternatives for raw materials, fabricators, or suppliers early in the design process. In a crisis, multiple fabricators who might normally compete may be willing to collaborate to increase production volume and provide complementary capabilities.

### Converting Needs Assessment Results to Technical Specifications

Requirements identified via needs assessment must be converted into functional and technical specifications that guide design. For example, if an end-user needs a face shield that is adaptable to different individuals, the functional requirement is for a product that fully covers faces of different sizes and has adjustable straps and attachment hardware. The technical specification might then be a shield of length of 22.5 to 30 cm and headband circumference of 50–60 cm. Analogously, an end-user requirement for reusability triggers a requirement for input from infection control experts and results in a functional specification for sterilizable designs and materials. The technical specification would then call for materials compatible with sanitizing wipes or hydrogen peroxide sterilization and an absence of crevices that can trap contaminants.

## STAGE 2: SOLUTION DEFINITION, VERIFICATION AND VALIDATION

Solution definition, verification and validation is an iterative process in which functional and technical specifications defined in Stage 1 are transformed into actual designs. Designs are then compared to specifications to verify that all requirements have been met. General considerations applicable to all medical products must also be included, such as the biocompatibility of materials in contact with humans. During a crisis, high demand for some types of equipment and raw materials may impose additional requirements on device components and processes.

### Rapid Manufacturing Techniques

Rapid manufacturing methods have a well-established role in facilitating rapid cycles of design and testing to reduce uncertainty and shorten production timelines (28). The use of rapid manufacturing methods is increasing in healthcare, facilitated in part by a series of FDA workshops (29). In the case of prosthetics (30), orthotics (31), tools for surgical planning (32), and dental and surgical equipment (31, 33, 34), additive manufacturing has opened new possibilities for designing products with complex geometries and allowed manufacturers to move away from providing only standardized products in a few sizes toward custom, patient-matched products. During the COVID-19 pandemic, additive manufacturing has been widely used to make face shields (31, 35, 36), nasopharyngeal swabs (37–39), face mask brackets (22), components for portable-air purifying respirators (PAPRs) (35), and ventilator splitters (40). In response to this activity, the FDA has released relevant guidance (3). Table 1 describes key manufacturing methods that are suitable for the production of substitutes for medical devices currently in short supply. The methods described use machinery that is available in both commercial (industrial) and consumer (maker) grades; however, industrial machinery is more precise, faster, and can typically process larger size products or materials. In many cases, designs prototyped on consumer-grade equipment can be successfully transitioned to higher-capability industrial machines.

Rapid manufacturing is used infrequently for PPE under normal circumstances primarily because the approach is typically more expensive (per unit) than conventional, large-scale production using methods such as injection molding. The limited production volumes of rapid manufacturing also pose a significant challenge to meeting the large demand created by the pandemic. Thus, they are most effective as a means for prototyping and short-term production or highly-distributed production, while conventional large-scale production methods ramp up.

### Sterilization and Reuse

To address acute shortages in devices that are traditionally single-use, such as respirators or face shields, the CDC issued guidance allowing for extended-use, reprocessing, and reuse of PPE (42). According to FDA regulations, hospitals and third-party reprocessors are considered “manufacturers” of the reprocessed devices and must comply with the same regulatory requirements as the original equipment manufacturers (43). Given the difficulty of compliance, the FDA and CDC guidelines have been relaxed for single-use devices during the pandemic. However, designers must take the necessary steps to ensure the compatibility of their devices with anticipated sterilization, decontamination, and cleaning procedures. This usually requires consultation with hospitals’ infection control experts as well as empirical testing. Additionally, fabricators should take the appropriate steps to ensure initial disinfection and sterilization of their products prior to delivery to end users. For devices such as faceshields, wiping down newly-fabricated units with approved disinfectants is most likely to be the appropriate procedure. The EPA provides a detailed record of products meeting criteria for use against SARS-CoV-2, along with corresponding directions for use (44).

In a pandemic, many products that are normally disposable end up being reused because they are in short supply. It should therefore be assumed that face shields, masks frames, and other items will be sterilized or decontaminated if at all possible. Sterilization methods of autoclaving, applying bleach-containing solutions, and alcohol-based wipes are widely available in healthcare and may be suitable for sterilization of products such as face shields. However, such methods often degrade key components, damage labels and safety warnings, and are not compatible with products such as N95 masks. Short wavelength ultraviolet (UV) light (45), vaporized or ionized hydrogen peroxide (46–48), and moist heat are more generally applicable but less widely available alternatives currently being developed for sterilization of masks and similar devices (45). Early into the design process it is important to determine which sterilization methods are available for testing and possible use with deployed products and then ensure their compatibility with a proposed product. The use environment should also be taken into consideration; for example, access to the sterilization equipment or decontamination solutions may be limited during a crisis and the process of getting products to and from centralized sterilization facilities must be considered. In many cases, the fundamental desirability of product reuse runs up against practical challenges with logistics. This is particularly true in the case of PPE that needs to be sterilized or decontaminated and then returned to the original users. We have found that many healthcare providers have been unable to put the necessary tracking procedures in place to make “return to original user” possible.

### Biocompatibility

Biocompatibility is defined by the FDA as the “ability of a material to perform with an appropriate host response in a specific situation” (49) where response refers to a host immune or inflammatory reaction to the material. Evaluation of biocompatibility is one part of the FDA’s overall determination of safety and effectiveness for new or modified devices that come into direct or indirect contact with the human body (50). In the US, two documents outline standard biocompatibility testing: the International Standard ISO 10993–1 (51) and the guidance related to ISO 10993–1 (49). A separate biocompatibility standard exists specifically for respiratory devices: ISO 18562–1:2017 (52). Among other factors, a biocompatibility assessment focuses on: (1) material chemistry and any changes caused by the manufacturing process, (2) material physical properties, (3) nature of the body contact (direct or indirect), (4) contact duration, and (5) prior history of safe use [as defined in ISO 10993–1 (49)].

Biocompatibility requirements for materials are application-specific and vary greatly based on the part of the body in contact with the device and the duration of contact; sustained internal contact is substantially more problematic than brief or external contact with the skin. Thus, approval of a material in one application does not constitute approval for another application. In addition to material considerations, the method by which a material is handled or processed during manufacturing may influence its biocompatibility. During biocompatibility evaluation, testing is performed on the “final finished form” of the device, which includes all manufacturing processes including packaging and sterilization. Rapid prototyping can be advantageous in conducting biocompatibility testing early in a product development cycle.

From a practical standpoint, most devices being subjected to rapid fabrication for pandemic response are for external use only and primarily contact the skin (or hair). In this setting, it is reasonable to use materials previously shown to be safe, such as silicones, parylene coatings, and many common fabrics. Particular attention should be paid to foam, elastic materials, and adhesives with respect to skin contact and latex should always be avoided; Monprene^®^ (PR-23040) is an FDA-approved alternative elastic material that is widely used in phlebotomy and is readily available.

**FIGURE 2 |:**
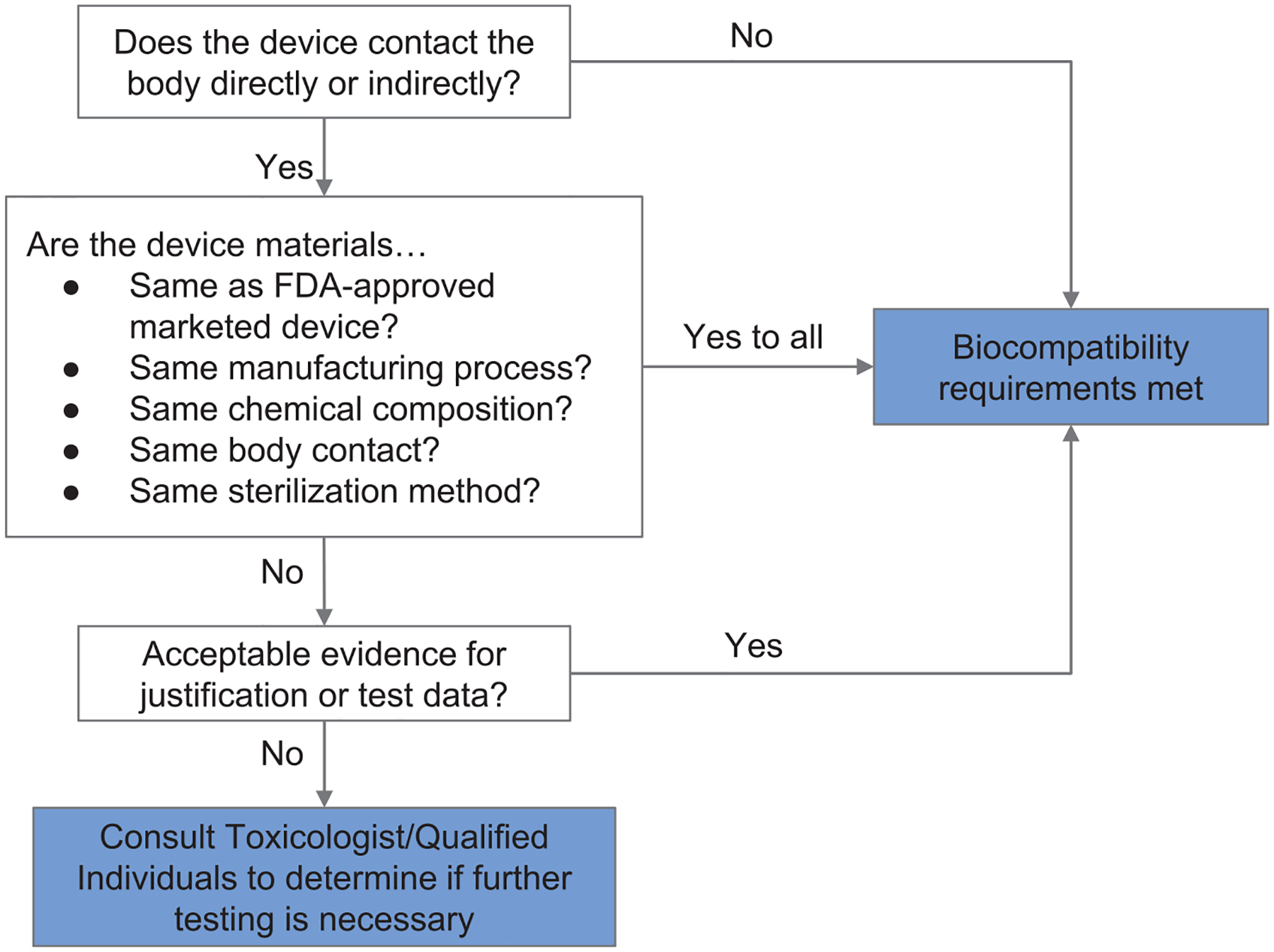
Systematic approach to a biological evaluation of medical devices as part of a risk management process. The process is simplified from FDA Guidance document for ISO 10993–1 (49).

If materials previously documented to be biocompatible in a particular setting are unavailable or functionally unsuitable, then biological endpoints ranging from cytotoxicity and sensitization to material degradation and carcinogenicity must be considered. Attachment A of FDA’s guidance related to ISO 10993–1 provides a list of the recommended biological endpoints to consider for the development of biocompatibility evaluation as well as the rationale for these endpoints (49). The flow chart of Figure 2 illustrates how one might evaluate biocompatibility to determine if a newly-developed device requires supplementary testing. During the current crisis, safeguards have been relaxed by regulatory authorities to enable more rapid response, so long as a specific medical device’s product code is explicitly mentioned in an FDA guidance or enforcement policy. These guidance documents are freely available on the FDA website (53).

### Design Verification and Validation (V&V)

Design verification is an iterative and empirical process in which objective evidence is sought to assess whether a product satisfies specifications. Design validation is a summative exercise that assesses the integrity of the final product and ensures that it meets user needs in a real or simulated use environment via testing, measurement, and observation of user interaction [commonly to ISO 13485:2016 (54)]. It should be noted that a product’s packaging, labeling, and instructions are considered to be essential parts of the product. Even in a crisis, it is important to provide inserts and labels with products describing their intended use, composition, and regulatory compliance (e.g., reference to an EUA).

### Scaling Up

While rapid manufacturing techniques such as 3D printing and laser cutting are efficient approaches for fabricating prototypes, low throughput and high unit costs do not make them feasible for large-scale production. In a traditional waterfall process, once a design converges on a final set of specifications, design transfer takes place. During the design transfer, the prototype design is adapted to the demands of large-scale manufacturing methods such as injection molding and die cutting. These methods are high-throughput and inexpensive per piece but are associated with significant up-front cost and set-up time (up to several months). Moreover, highly specific expertise is generally required to make design dies and molds compatible with a specific type of equipment. In a crisis, in which time is usually limited and production costs are less of a concern, parallel production using the prototyping facilities of many manufacturers, colleges, and makers can be a good way to meet demand. For example, in the case of the Panfab/BWH face shield (Box 1), 3,000 face shields were fabricated in a few weeks using 3D printing and laser cutting at multiple sites (36) and the Czech 3D producer PRUSA has described a highly parallel face shield printing process using inexpensive machines (35).

Conversations with suppliers and manufacturers will help guide prototyping and design processes and prepare for design transfer to a manufacturer, if relevant. A responsible entity may be required to register with the FDA as a manufacturer and list the product(s) they are distributing or selling, or the services they are providing to other manufacturers, depending on the product code, respective risk classification, and regulatory compliance pathway (55). Meeting these requirements will be especially important once the public health emergency has ended. Additional stakeholders such as payers and post market surveillance organizations may also come into play (Supplementary Figure 1). It is currently unknown whether traditional manufacturers will adopt some of the innovative designs developed by non-traditional suppliers during the current pandemic and shepherd them through the regulatory process. We hope that this is the case, but much depends on the creation of better market incentives (see below).

## STAGE 3: REGULATORY APPROVAL AND IMPLEMENTATION

On January 31, 2020, the US HHS Secretary Alex Azar declared a public health emergency involving COVID-19, noting that the circumstances justified emergency use of *in vitro* diagnostics and other medical devices that aid in the detection or diagnosis of COVID-19 (56). Pursuant to this declaration, the FDA has issued Emergency Use Authorizations (EUAs) for a number of medical devices. As mentioned above, EUAs allow certain non-FDA approved medical products to be used in the absence of adequate FDA-approved alternatives (57). While some EUAs are manufacturer-specific, others are broader in scope. For example, EUAs for face shields and respirators waive certain FDA requirements for all prospective manufacturers, authorize the modification of approved products, and allow for extended use, widespread production, and distribution of devices so long as manufacturers adhere to requirements outlined in the EUA notice (58, 59). EUAs expire upon resolution of the public health emergency. The agency has also provided extensive and frequently-updated guidance documents for manufacturers seeking to produce rapid diagnostics, personal protective equipment (PPE), and other devices for front-line use in responding to COVID-19. EUAs have also extended into the realm of sterilizing PPE for re-use.

A lack of strict regulatory oversight does not absolve designers and fabricators from doing their best to ensure that products are safe and functional and do not put healthcare providers or patients at risk. Additionally, disclosure of risk to the end-user is important: the FDA EUAs and enforcement policies include specific requirements for labeling, information for use, and use environments to ensure that products do not create undue risk.

### Overview of Medical Device Regulatory Review and Implementation

In the US, medical devices are typically made available via the FDA’s 510(k) premarket notification process. Depending on the degree of risk associated with the use of a medical device it is classified by the FDA as either Class I (low risk), Class II (moderate risk), or Class III (high-risk) (60, 61). The 510(k) submission process requires that a manufacturer of a device new to the market demonstrate “substantial equivalence” to one or more legally marketed devices, thereby avoiding a requirement for extensive clinical testing. Substantial equivalence does not require that a device be identical, but instead mandates that it be as safe and effective as an existing marketed device (62). Some Class I products are exempt from the 510 k premarket submission process based on the evaluation that these products pose a particularly low risk. This includes devices such as face shields, most forms of PPE, and nasopharyngeal swabs. Class II devices include more complex life-critical products such as ventilators and Class III devices traditionally encompass products implanted into a person, such as pacemakers, defibrillators, or prosthetics. Almost all devices currently being supplied by non-traditional fabricators and maker communities fall into Class I, and the majority are Class I exempt. However, ventilator splitters, which have been much discussed as a way of increasing ventilator capacity (63), may be Class II devices (64) and considerable controversy has attended their development (65).

The premarket submission process only clears a manufacturer to market a product; additional requirements must be met prior to manufacturing, selling, or distributing it. Quality controls in manufacturing, referred to as good manufacturing practices (GMP), include a requirement of product traceability in case of manufacturing flaws or product recalls. Notably, no Class II devices, and only a small number of Class I 510 k exempt devices, are also exempt from GMP regulations (66). Meeting GMP standards constitutes a substantial barrier for formal certification of devices fabricated through local, nontraditional manufacturing practices.

After implementation, unique device identification, an important part of the FDA’s post-market surveillance process, ensures that manufacturers vigilantly and proactively monitor the use of their product(s) for adverse events and patient injuries. The post-market surveillance process can also act as means to collect user feedback, which can lead to product improvements or innovative alternatives to existing designs. In the current crisis, US regulators have put in place emergency authorizations that waive some of the normal GMP and tracing requirements in favor of less stringent discretionary enforcement. In many cases, this forms the regulatory foundation for repurposing products from the non-medical supply chain and for hospitals to engage with informal networks of maker communities and similar organizations (67). The underlying logic is that a more permissive stance on low-risk Class I products better balances the hazards associated with a lack of supply against the hazards of using new designs and non-traditional manufacturing processes.

The use of research protocols under the purview of Institutional Review Boards (IRBs, similar to ethics committees outside of the US) provides one well-established route for testing new products in a clinical setting. Risk assessment, informed consent, and certification of human subjects training are standard components on an IRB submission, which should be familiar to many investigators in academic medical institutions. Collaborations between such individuals and maker communities or private fabrications are therefore encouraged. While, private “pre-certification” laboratories that perform testing of devices to prevailing regulatory standards in the US, Europe, and Japan can provide a substantial degree of confidence in a non-traditional product, they may still require GMP certification for use under the FDA guidelines.

### Product Safety Validation for PPE

Product safety validation for PPE and similar low-risk devices is commonly performed by certified testing laboratories, many of which are commercial and provide fee-based testing for compliance with specific regulatory standards (e.g., NIOSH standards for PPE). Under normal circumstances, regulations require that quality management systems (QMS, e.g., to ISO 9001) be in place. A QMS specifies how design and development inputs and outputs should be documented, how records should be maintained on the skills, the experience and qualifications of key personnel, and how calibration records should be established and monitored. Statistical Process Control (SPC) is also widely used throughout medical device manufacturing (and manufacturing in general) to ensure product consistency and quality. In a crisis-responsive setting, QMS and SPC are likely to be infeasible, which makes formal certification by NIOSH or FDA standards impossible. In such instances, makers must do their best to maintain quality and test either independently or via the use of other local resources such as academic laboratories. Consortia such as the Mass General Brigham Center for COVID Innovation (68), N95Decon (69), and M-ERT (17) may be able to provide guidance in specific situations.

### Use of Research Protocols

An effective and well-established way to test non-traditional medical products in a healthcare setting is to use research protocols overseen by an Institutional Review Board (ethical review board; IRB); these are part of the normal operation of virtually all teaching hospitals. The use of a research protocol makes clear to participants, via the process of informed consent, that a product is being tested and that it has not necessarily been through the usual regulatory review. IRB approval is also almost always required for conducting user surveys and receiving direct feedback from end-users. Devices such as face shields (described in Table 2) are FDA regulated Class I products with the lowest risk to human health. They are therefore suitable for testing in an IRB-supervised research protocol under even the suboptimal conditions of a healthcare emergency. We have found that the use of research protocols for product testing increases buy-in from healthcare leadership, in part because it is a familiar process. In the case of a non-traditional face shield as described in the case study below (Box 1), it was possible to perform IRB protocol review and product specification, validation, and testing in a clinical setting in a period of roughly three weeks (36). IRB review was performed in less than one week as a result of specific steps put in place by area hospitals to accelerate the introduction of COVID-19 related protocols. More commonly, IRB review for non-invasive, low-risk protocols takes several months.

## GENERAL RECOMMENDATIONS

A crisis-responsive design framework aims to couple user needs, rapid manufacturing technologies, and local fabrication to fulfill unmet demand for simple medical devices in times of crisis. Below we provide three sets of recommendations intended to serve as a checklist for (1) designers, maker communities and fabricators, (2) healthcare providers and administrators, and (3) regulatory bodies overseeing pandemic response. Note that detailed regulatory documents from NIOSH, ANSI, ISO, and DIN typically cost several hundred USD but many are being made freely available for the duration of the current healthcare emergency.

### Recommendations for Designers, Maker Communities, and Fabricators

1. **Conduct a thorough needs assessment** to understand the key features and requirements for the proposed product and its labeling, assess likely demand from different types of users, establish requirements for sterilization or decontamination, secure appropriate materials and mitigate any supply chain issues (e.g., by using alternative raw materials), and establish tolerances for different styles and types of products. Perform this analysis by speaking to a broad range of stakeholders early in the process and make sure to consider diverse body types and clinical roles. Secure examples of existing products and assess their strengths and weaknesses. Download and review relevant NIOSH/ANSI or regulatory documents from other agencies.
2. **Solicit end-user, expert, and clinical feedback early and often** throughout the design process to ensure the product satisfies anticipated and unanticipated needs. Frequent assessment increases the likelihood of end-user buy-in and enables rapid adoption of new solutions in response to changing demands. Consulting with senior medical staff and division heads can be helpful in this setting. Establish a testing process and determine whether an IRB-approved clinical protocol may be required; it is necessary if a formal survey is to be conducted.
3. **Conduct a rigorous assessment of biocompatibility, sterilization, and risk**. For PPE-type devices, research issues related to biocompatibility, manufacturing, intended duration/frequency of use, and anatomic location. Use materials previously established to be safe for the intended use if at all possible. This assessment is usually based on the scientific literature, medical device standards, or data on devices previously reviewed by the FDA. Be aware of likely sterilization requirements and feasibility since not every method of sterilization is compatible with every device. Review ISO 14971 (55), ISO 10993–1 (51) and FDA’s associated guidance (49), and ISO 18562–1 (52) for additional information. Determine if proposed sterilization methods will in fact be available for testing and use when products are deployed.
4. **Search design repositories for suitable designs** that can be used as-is or modified to meet local requirements and fabrication capabilities. Many online forums have emerged to assist in the dissemination of best practices. Publicly funded designs should be available under nonrestrictive licenses from resources such as the National Institutes of Health 3D Print Exchange (4).
5. **Consider manufacturing methods**, such as 3D printing and laser cutting, which enable rapid prototyping, and low-volume manufacturing, while keeping in mind a possible transition to other approaches (e.g., injection molding) for large scale manufacturing. Consider which types of equipment will be available for the duration of the project and whether local shops can be contacted for access to higher-end equipment.
6. **Avoid action bias**, which results in premature action and over-rapid development of potentially suboptimal or undesired solutions. Even in a crisis, it is important to proceed deliberately to ensure that products are usable, safe, and durable.
7. **Develop a written process for device performance validation** through measurement of fit-for-purpose criteria and alignment with end-user needs and regulatory standards. Check with target users regarding the testing requirements and IRB-based testing capabilities. Consider life cycle issues including whether products will be withdrawn from service at the end of a medical emergency.
8. **Provide documentation.** Make sure to include accurate and complete labels and product information as inserts with the final products; consider complementing this with QR codes and online resources to provide users with the most up to date information. Consider making new designs and any improvements on existing designs publically available.
9. **Perform multiple activities in parallel.** Given the need to provide products as rapidly as possible in a pandemic, processes that are traditionally performed in a sequential matter should be parallelized to the greatest extent possible. Needs assessment, literature review, and IRB submission can occur in parallel. Collecting information on biocompatibility, sterilization, and risk can be started based on some assumptions about materials. Once a prototype is available, input from potential users can be solicited. Note however, than any data collection via surveys or field studies requires IRB approval so securing this approval is a critical step and potential bottleneck. At this time potential fabricators and sources of material can be lined up. However, before proceeding to actual manufacturing, test data must be analyzed and a final validation assessment performed. This is a second critical step and the information should be reviewed by the design, clinical, and fabrication team members. Labels and product documentation must be completed before devices can be delivered to the end user and all products must be decontaminated or sterilized; this is the third critical step in the path to providing useful products into a healthcare setting.

### Recommendations for Healthcare Providers and Administrators

1. **Assemble a diverse crisis response team.** Healthcare providers should add one or more individuals with experience in manufacturing, product design, or with maker communities to incident command and crises response teams. These individuals should be charged with outreach to non-traditional suppliers and emerging resources (e.g., the 3D Print Exchange) prior to a crises. In many cases it will be possible to identify individuals with valuable expertise in clinical teams. The engineering organizations in many hospitals can also be helpful. A diversity of perspectives and providing key personnel with the time and resources to understand regulatory documents associated with non-traditional fabrication is essential for sourcing non-traditional medical supplies.
2. **Develop more robust supply chains.** Health care providers should consider the resilience of supply chains in addition to cost. The likelihood that all suppliers will simultaneously be unable to supply key products must be considered, since the number of original equipment manufacturers is often much smaller than the large number of branded products would suggest.
3. **Use IRB Review Process to test non-traditional medical supplies.** Using expedited IRB review is an effective way to test non-traditional medical products in a healthcare setting. However, it is necessary that IRBs work efficiently under emergency circumstances when low-risk Class I and exempt products are being tested.

### Recommendations for Regulatory Bodies

1. **Maintain current regulatory standards under normal conditions.** We do not propose that regulatory standards for PPE and simple medical products be relaxed, but consideration must be given to foster innovation. Federal reports have repeatedly identified deficiencies in existing PPE but few if any improvements have been made in designs or supply chains. This situation should be rectified before the next emergency and may require government funding.
2. **Develop policies that facilitate fabrication of high quality non-traditional products** for use specifically in medical emergencies. These policies might reasonably include some of features of a crisis-responsive framework outlined above as well as pre-tested public domain designs and prescriptive fabrication approaches.
3. **Increase the transparency of Emergency Use Authorizations to improve submission and approval.** The minimum standards needed for submission of a request for an FDA EUA for new technologies must be improved. As it currently stands, it is difficult to ascertain key features of products in the non-traditional supply chain from commercial manufacturers. For example, the FDA authorized distribution of foreign manufactured N95-style masks and did not require that companies provide basic operational data including name and place of business, proprietary or brand name, model number, marketing authorization, and a copy of the product labeling (81). At the very least, data submitted to the FDA in support of an EUA should be made public to the greatest extent possible.
4. **Provide an accelerated pathway to transition products authorized under an FDA EUA to either traditional or emergency-only approval.** It is highly desirable that innovative designs and approaches developed during the pandemic be further developed, tested, and integrated into normal supply chains so that they are ready for future emergencies. In the US such a task falls outside of the remit of regulators such as the FDA and NIOSH but might be tackled by the Dept. of Health and Human Services or even the Department of Defense. Since PPE for pandemic response is intended to be a public good, international cooperation would be highly desirable.

## CONCLUSIONS AND FUTURE PROSPECTS

The COVID-19 pandemic has made clear the fragility of medical supply chains whose breakdown has resulted in rapid and severe shortages for many essential medical supplies. The communities of designers, fabricators, and healthcare professionals who have come together to supply locally made substitutes using rapid manufacturing methods have revealed a hitherto untapped capacity to make the provision of medical supplies more resilient. The great strength of community efforts is that they avoid extended product development cycles and bypass international lowest-cost production in favor of rapid innovation and efficient execution. However, even in a crisis, the risks of using alternative, locally-manufactured medical devices must be carefully evaluated and mitigated. Hastily designed products made without appropriate stakeholder input are unlikely to be used clinically, representing a loss of time and resources by well-intentioned makers. If poorly designed devices make their way into clinical use, they can pose a substantial hazard. This perspective provides a crisis-responsive framework for medical product design that is intended to avoid these risks. The framework is informed by traditional FDA guidance on product approval, implementation, and validation, but adapt that guidance to accommodate constraints in time, material resources, and human capital.

What does the current state of the COVID-19 response portend for the future? From the perspective of nontraditional fabricators of low-risk medical devices and PPE, a transition from purely local efforts to national and international collaborations is already underway. These initiatives include several efforts focused on PPE including Get Us PPE (82), Covid19 Masks (83), Mask Match (84), Project N95 (85), and PPELink (86) and larger efforts such as Open Source Medical Supplies (OSMS) (87), America Makes (19), and the NIH 3-D Print Exchange (4). State governments and companies such as Gillette (a P&G Company) (88), and Lovepop (89) are coming together through initiatives like the Manufacturing Emergency Response Team (M-ERT) (17) to design, develop, manufacture, and donate or sell thousands of devices. These consortia facilitate stakeholder interactions and device testing, operations that are hard to perform on individual bases. Continuing these efforts will build greater resilience for future pandemics.

A key unanswered question is what will happen to non-traditional medical devices and innovative designs when the COVID-19 pandemic recedes. It is possible that even the best innovations will be abandoned in favor of a return to standardized lowest-cost alternatives. The design deficiencies of these existing products and the fragility of supply chains have long been known and analyzed in a comprehensive series of government reports spanning two decades, several of which were undertaken in response to MERS, SARS, influenza, and other zoonotic transfers. A wide variety of potentially effective solutions were proposed by government and private entities (67) but few were actually implemented. A key problem is the high pressure on costs, which for commodity products inevitably results in the use of lowest cost suppliers and products that barely meet operational requirements. The current pandemic has revealed the weaknesses of this approach, but we have witnessed little discussion about the necessity of paying more to maintain supply chain resilience.

To avoid forgetting the lessons of COVID-19 pandemic, it is essential that innovative designs and approaches be further developed, tested, and integrated into normal regulatory chains so that they can be used in standard products or readied for future emergencies. Changes in regulatory policies are needed to balance the rigidity of the pre-crisis approach with counterfeiting (81) unintentionally enabled by EUAs. We envision the development of open-source repositories, such as NIH 3D Print Exchange, to facilitate independent testing of products that meet key NIOSH, FDA and other requirements for functionality and safety. These should be accompanied by simple prescriptive approaches and best practices geared to the capabilities of small-scale fabrications. Such guidance could mimic the practice in building codes [e.g., those from the International Code Council (76)] of providing both a limited number of pre-engineered solutions that are ready for application in the field while also allowing for a wider range of solutions when engineering resources are available. In a crisis, guidance of this type would take the place of QMS and SPC and improve products even when the requirements for GMP cannot be met. Training materials for designers, fabricators, and healthcare institutions should be also developed to help break down barriers to communication and should be placed in the public domain rather than behind paywalls. Institutional changes should also include adding makers and engineers to incident command and procurement teams so that non-traditional designs can be more effectively vetted; model IRB protocols should be prepared for products that need clinical testing. Finally, patented designs should be placed in repositories for public use during health emergencies (67) to avoid delays from patent disputes. Such improvements to our infrastructure will dramatically improve our ability to respond to future medical pandemics and medical emergencies in the US and across the globe.

## Supplementary Material

Supplementary Material

## Figures and Tables

**FIGURE 3 |:**
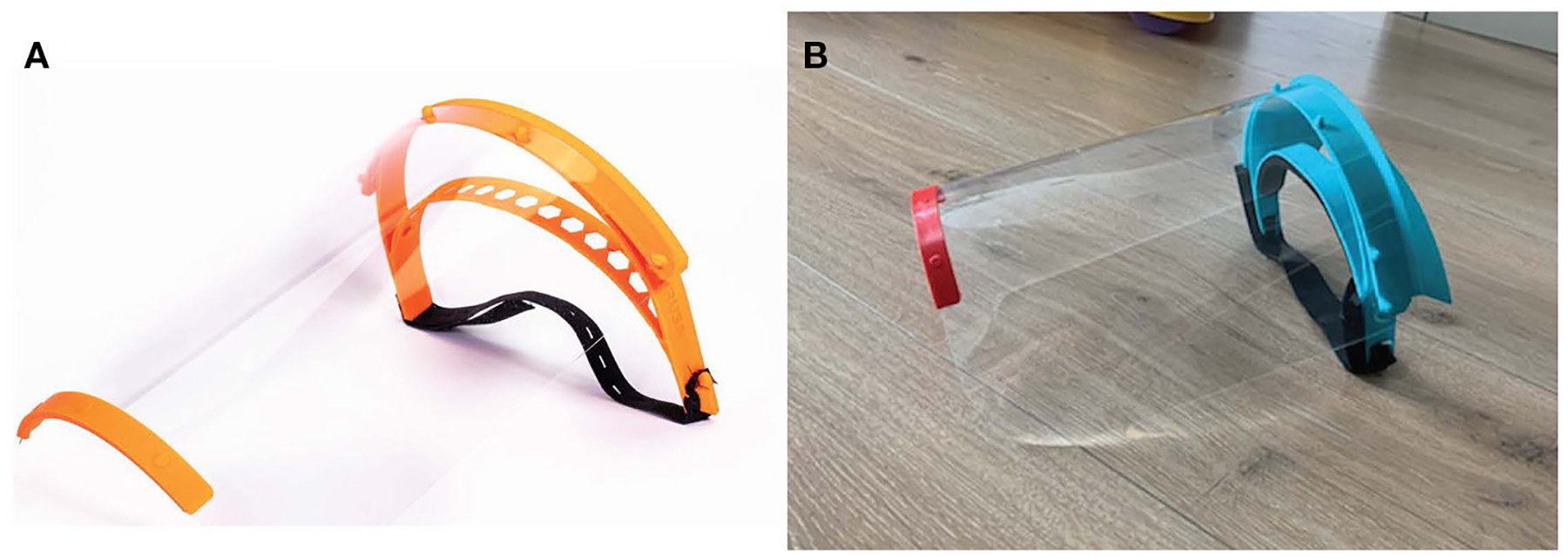
Face shields fabricated during the COVID-19 pandemic. **(A)** Image of Prusa RC2 design and **(B)** final PanFab face shield prototype. See text for references and details.

**TABLE 1 |:** Prototyping and manufacturing methods applicable to production of five medical devices, based on Open Source COVID-19 Medical Supply Guide (41).

Categories	Set-up cost and time	Prod. cost and time	Specific methods	Face shields	Nasapharyngeal swabs	Surgical face masks	N95 respirators	PAPRs	Ventilator splitters
Rapid Prototyping	Low	High	3D Printing (FDM)	Yes	Yes	No	Yes	Yes	Yes
			3D Printing (SLA)	Yes	Yes	No	Yes	Yes	Yes
			Machining	Yes	No	Yes	Yes	Yes	TBD
			Laser Cutting	Yes	No	Yes	No	Yes	No
High Volume Production Processes	High	Low	Die Cutting	Yes	No	Yes	Yes	Yes	No
			Injection Molding	Yes	Yes	No	Yes	TBD	Yes
			Compression Molding	Yes	Yes	No	No	TBD	TBD
			Thermoforming	Yes	Yes	No	No	TBD	TBD
Fabrication and Assembly	Variable	Variable	Sewing	No	No	Yes	Yes	Yes	No
			Gluing and Bonding	Yes	Yes	Yes	Yes	Yes	TBD
			Fastening	No	No	No	No	Yes	No
			Electronics	No	No	No	No	Yes	No
			Assembly	Yes	No	Yes	Yes	TBD	Yes

**FDM**: Fused Deposition Modeling. An additive manufacturing (3D printing) process that can be implemented using low-cost consumer-grade equipment. A printer heats up a plastic in filament form and uses a print head to create a 3D structure from the bottom up.

**SLA**: Stereolithography. Another additive manufacturing process using low cost consumer-grade equipment in which a vat of liquid resin is hardened with a laser to create desired shapes.

**Machining**: Conventional (subtractive) manufacturing in which raw material is cut into a desired shape by a controlled material-removal process such as milling and routing. Small, low-volume, computer numerical control (CNC) machines are increasingly widespread.

**Laser cutting**: A technology in which a high-powered laser is used to cut sheet materials to size; consumer-grade laser cutters for plastics are increasingly inexpensive.

**Die cutting**: A technology in which metal knives (dies) with sharp edges are pressed into sheet materials to cut them to size and shape. Production time per piece is significantly faster than laser cutting but requires more expensive and longer setup due to the custom-fabrication of the die.

**Waterjet cutting**. A technology in which a narrow jet of high pressure water and abrasive is used to cut sheets of material; desk-top waterjet machinery is now available.

**Injection Molding**: A high-volume manufacturing technology that produces parts by injecting molten material (polymers, glass, metal) into a mold. The upfront cost and setup time of injection molding is high but unit cost and production time are low.

**Compression molding**: A system by which heat and pressure are applied to a material in order to shape it.

**Thermoforming**: A technique by which plastic is heated, formed to a specific shape in a mold, and trimmed to create a desired shape.

**TABLE 2 |:** Summary of regulatory and testing standard for each device.

Medical supply	Device FDA product code	Traditional regulatory process	Revised process during COVID-19 pandemic	Standards for testing (NIOSH/ANSI)
Face shield	LYU	FDA regulated Class I 510(k) exempt	FDA Emergency Use Authorization (EUA) (58) FDA Enforcement Policy	ANSI/ASSE Z88.2–2015
Nasopharyngeal (NP) swabs	KXF, KXG	FDA regulated Class I 510(k) exempt	No EUA	Not applicable. See text for description of materials, PCR compatibility, transport media compatibility, mechanical performance, and length considerations.
Surgical face masks	FXX	FDA regulated Class II 510(k) (70)	FDA Enforcement Policy (71)	6–254 ASTM F2100–11; 6–335 ASTM F2101–14; 6–406 ASTM F1862; 6–425 ASTM F2100–19; 6–427 ASTM F2101–19
N95 respirator	ONT, ORW, NZJ	FDA regulated Class II 510(k) clearance	FDA Emergency Use Authorization (EUA) (59)	NIOSH (72, 73)
PAPR	N/A	NIOSH regulated, FDA approved (74)	FDA Emergency Use Authorization (EUA) (59) as a subset of filtering facepiece respirators (FFRs) (75)	NIOSH (76)

* Only true for the duration of the crisis. These EUA guidelines are up to date as of the time of submission.

## BOX 1 | Case study: nontraditional design and fabrication of face shields during a health care emergency.

### A Case Study on Fabricating Face Shields in the Northeast U.S.

In response to the COVID-19 pandemic, which grew rapidly in Massachusetts during March 2020, multiple teams formed to address rapidly growing shortages in medical supplies. Some of these teams were established by institutional mandate and others arose spontaneously through the efforts of engaged individuals. An example of the latter is the Greater Boston Pandemic Fabrication Team [PanFab (20)], a student-faculty initiative organized by the Harvard-MIT Center for Regulatory Sciences. It consists of a group of volunteers with expertise in engineering, biomedicine, manufacturing, and regulatory review working closely with the physicians at Boston-area hospitals, the local maker community, and manufacturing experts from local companies contributing outside of normal work hours. A physician assigned to a hospital incident command was a particularly important member of the PanFab team because she could provide timely and accurate information on current and emerging needs. PanFab has been effective in designing and rapidly fabricating face shields, mask frames, PAPRs, and other types of PPE (36) and its activities are representative of local design in response to a healthcare crisis.

#### Stage 1: *Problem Definition and Needs Assessment*.

##### Defining the problem

Faceshields are a critical component of PPE per the CDC (77) and are used in conjunction with surgical masks or N95 FFRs to protect the face and neck, particularly mucous membranes in the eyes, nose, and mouth, from splatter by contaminated bodily fluids (78). However, during the COVID-19 pandemic, a severe shortage of face shields developed. Face shields are composed of a clear shield (commonly made from BoPET, PETG, acetate, or polycarbonate) and a headband frame that is in contact with a user’s forehead and commonly made from a lightweight plastic or foam (78). In a US health care setting, face shields are traditionally single use devices, but because of shortages they are being worn for the full duration of a shift (6 to 12 h) and then sterilized for reuse.

##### Needs assessment results and associated technical specifications:

From interviews with healthcare providers, hospital administrators, and infection control specialists, the following needs were identified:

1. The shield must protect mucosal membranes from splashes of bodily fluid by extending from the forehead to the points of both ears and down the neck.
2. The shield should not fog or otherwise obscure the user’s view, even during strenuous activity that may produce perspiration (78).
3. The shield should fit a range of facial lengths and heights while not interfering with range of head and neck motion.
  - Associated technical specification: facial lengths 23–30 cm and range of motion 180 degrees in each direction.
4. The shield should remain firmly in place and remain comfortable when the user moves their head up, down, and laterally at varying speeds.
5. Face shields are typically worn for the duration of a shift (up to 12 h). Thus, the shield cannot be so heavy as to cause discomfort when worn for a shift.
6. Attachment mechanisms should remain firm while preventing skin sensitization, irritation, or imprints on the skin. The attachment must be adjustable.
  - The FDA EUA allows specific materials to be re-used without thorough re-testing; these are materials of choice for the design.
7. Device cost in terms of materials should be <$5 per shield; design and fabrication time are to be donated.
8. Hospital demand requires a production rate of 2,000 shields per month.
9. The face shield should require minimal assembly, both for ease of use and to limit the presence of hard to reach surfaces, which could interfere with the decontamination process.
10. To be reusable, the face shield should be compatible with the hospital’s commonly used sterilization techniques, which include ionized hydrogen peroxide, germicidal disposable wipes, or 70% isopropanol wipes.

#### Stage 2: *Solution Definition and Validation*

##### Prototyping and Manufacturability:

Given the low-risk nature of the device, various 3D printing approaches were considered acceptable. We began with the open-source Prusa design (35) and iterated it based on feedback from healthcare providers at Brigham and Women’s Hospital (BWH) (Figure 3). The new design added a forehead fin with a drip guard that protected the otherwise-exposed forehead from body fluid exposure. This requirement was not identified during initial assessment but became obvious once prototypes were in the hands of emergency room users. The prototype shield was also too narrow and short and did not provide sufficient splash protection for the neck and sides of the face for all users; the length and width of the shield was therefore increased.

##### Sterilization and Reprocessing:

Given that safe sterilization of 3D printed face shields had not been extensively studied, we followed CDC’s guidance (79) relating to sterilization and reprocessing compatibility for goggles, a related product. We checked for changes in material properties and visor transparency following several days of use and following regular cleaning with sanitizing wipes (Germicidal Disposable Wipes). We also ensured that the face shield could withstand ionized hydrogen peroxide sterilization (iHP; TOMI SteraMist), resulting in effective killing of test bacterial spores as measured by standard biological indicators (80).

##### Biocompatibility

A number of materials were assessed during fabrication of the face shield based on (i) resource availability, (ii) previous uses in marketed and FDA-approved medical devices, and (iii) compatibility with common sterilization techniques. In the case of the face shield, only the 3D printed headband, the foam pad, and the Velcro strap of the face shield were in contact with user’s skin or hair. Applying the workflow shown in Figure 2, the requirement for safe interaction with intact skin was met based on limited duration body contact (< 24 h) and prior material biocompatibility information obtained from material Material Safety Data Sheets (MSDSs), reported experience from manufacturers, and published literature. PLA was selected as the material for 3D printing the headband because of its wide availability and because it is known to be safe in contact with the skin. The Velcro strap, which is made from polyethylene and nylon, is also known to be safe for skin contact. Similarly, we established that EVA foam was safe for skin contact. We selected closed-cell EVA over its open-cell counterpart—which is absorbent—and evaluated its compatibility with common sterilization and reprocessing techniques [70% isopropanol wipes, ionized hydrogen peroxide (80)].

#### Stage 3: *Regulatory approval and Implementation*

##### Regulatory approval:

Face shields are an FDA regulated Class I 510(k) exempt device (78) making them appropriate to attempt to fabricate locally. Additional information on the FDA regulation of face shield products during the COVID-19 pandemic can be found in Table 2.

##### Clinical Testing:

To assess face shield usability and safety, a cohort of 97 physicians, physician assistants, emergency department technicians, environmental service staff, and other individuals with patient-facing roles were recruited to an IRB-approved study from the Emergency Department at BWH. Users wore the face shield during their shifts and completed a questionnaire on baseline experiences and attitudes. A majority of users indicated that they had a better experience with the PanFab face shield as compared to the hospital standard-issue, disposable model. This data was then presented to the appropriate stakeholders at BWH, and the product received approval for use as part of a clinical workflow.

##### Production and Implementation:

Following validation of the final design through an IRB-approved study at BWH over 3,000 face shields have been manufactured and deployed to meet local demand. These face shields were produced via distributed rapid manufacturing in collaboration with a local makerspace community (BoroBot), Salesforce, a small-scale prototyping company (SunPe Prototype), large-scale manufacturers of non-medical supplies (iRobot and Velcro), in addition to academic partners (Harvard Graduate School of Design, the Shin Laboratory at the Brigham and Women’s Hospital’s Stepping Strong Foundation for Trauma Innovation, and the Wentworth Institute of Technology). All design files were made openly available on the PanFab website and on the NIH 3DPrint exchange platform to allow other manufacturers and localities to produce the face shield.

### Timelines and key dependencies

Once the need for an alternative source of face shields was established via consultation with hospital incident command, four individuals from the PanFAB group became engaged with the project. Problem definition and preparation of documents for the IRB occurred concurrently and required approximately two weeks. Following this, prototyping and soliciting user feedback on modified designs took another two weeks. User testing in a clinical setting was dependent on having IRB approval and a final design. Collecting and consolidating results took another week and was overseen by three physician-scientists. During this time, production materials were procured and four production sites were lined up. Production began when analysis of testing data was complete and first delivery of face shields occurred six weeks after the project started; production continued for six weeks at four sites, yielding a total of ~3,000 face shields.
