# Supplementary material for: A Crisis-Responsive Framework for Medical Device Development Applied to the COVID-19 Pandemic
Source: Front Digit Health. 2021 Mar 22;3:617106. doi: 10.3389/fdgth.2021.617106 (PMC8064560; doi:10.3389/fdgth.2021.617106)
Supplement: Supplementary file 1 [file Table_1.DOCX]

Supplementary Material


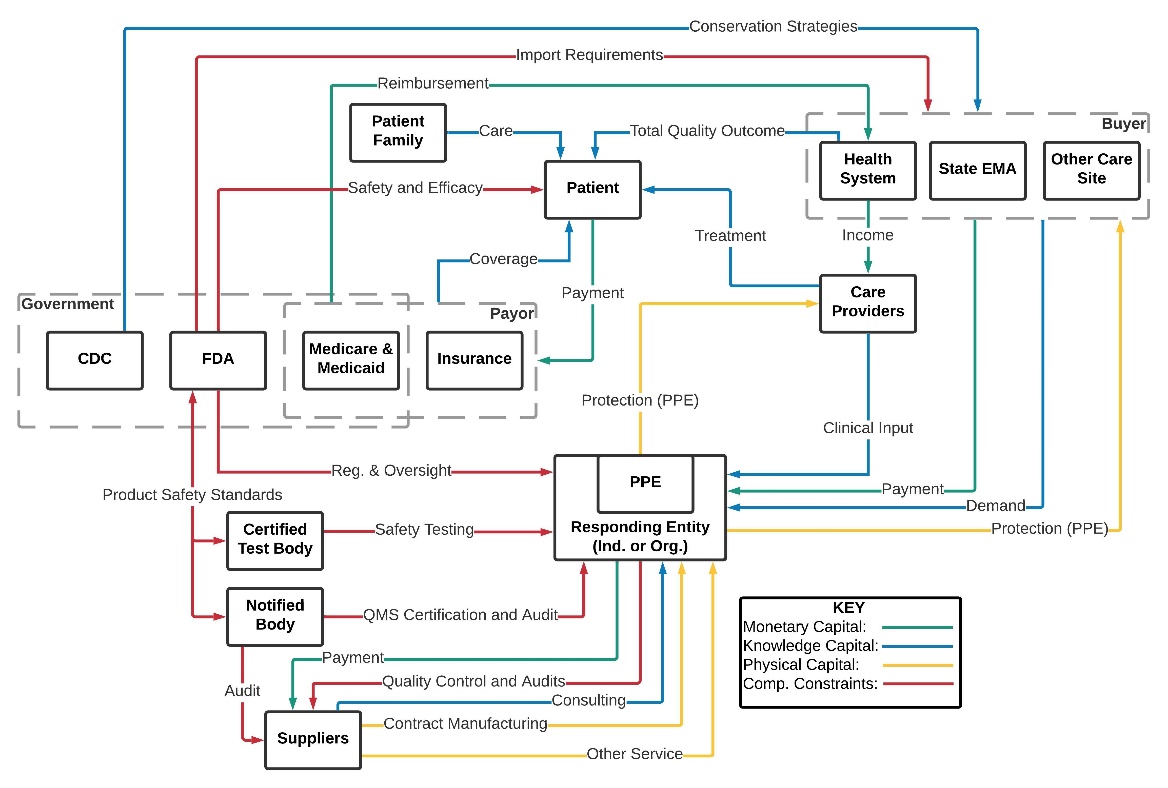


**Supplementary Figure S1: Stakeholder value network demonstrates the complex relationship between all the stakeholders involved in the conventional medical device process.** **Related to Figure 1.** Products scaling up must eventually consult and comply with the needs and regulations of all of these stakeholders. QMS: quality management system, Ind: Individual, Reg: regulatory, Org: organization. Comp: Compliance.
